# Warts

**Published:** 1916-10

**Authors:** 


**Warts.** Sulphur sub. 20%, concentrated acetic acid 10%, Glycerin to 100% is recommended for withering warts promptly, by the editor of the Surgical Clinic.

